# In vivo target protein degradation induced by PROTACs based on E3 ligase DCAF15

**DOI:** 10.1038/s41392-020-00245-0

**Published:** 2020-07-27

**Authors:** Liang Li, Dazhao Mi, Haixiang Pei, Qiuhui Duan, Xinyue Wang, Wenbo Zhou, Jianping Jin, Dali Li, Mingyao Liu, Yihua Chen

**Affiliations:** 1 Shanghai Key Laboratory of Regulatory Biology, Joint Research Center for Translational Medicine, ECNU-Fengxian Hospital, Institute of Biomedical Sciences and School of Life Sciences, East China Normal University, Shanghai, 200241 China; 2 Joint Center for Translational Medicine, Fengxian District Central Hospital, Shanghai, 201499 China; 3 MOE Key Laboratory for Biosystems Homeostasis & Protection and Innovation Center for Cell Signaling Network, Life Sciences Institute, Zhejiang University, Hangzhou, Zhejiang 310058 China

**Keywords:** Haematological cancer, Haematological cancer

**Dear Editor,**

As an emerging drug discovery paradigm, Proteolysis targeting chimeras (PROTACs) facilitate ubiquitination and degradation of the targets by cellular endogenous proteasome system. Compared with traditional small molecular inhibitors, PROTACs work like enzymes and can operate with or without functional inhibition of the targets, which endows this strategy to degrade drug targets, especially useful for “undruggable” ones.^1^

Despite the advantages and broad scope of successful target degradations, the narrow pool of well-characterized E3 ligases has so far limited the advancement of PROTACs in these applications. Although there are more than 600 E3 ligases encoded by human genome, only a few have been successfully hijacked by present PROTACs, such as VHL, CRBN, MDM2, and cIAP. Several studies have revealed that off-target effects and resistance mechanisms emerged in cells following the treatment of VHL- or CRBN-based PROTACs.^1^ Therefore, exploring the ‘PROTACs-ability’ of other E3 ligases with different properties could facilitate the pool of effective PROTACs. Recently, it’s reported that antitumor sulfonamides can recruit DCAF15 E3 ligase to target the splicing factor RBM39 for ubiquitination mediated proteasomal degradation.^2^ In addition, novel electrophilic PROTACs based on DCAF16 were reported.^3^ As a member of the same subfamily of DCAFs, DCAF15 has been recently proposed in PROTACs design by Zoppi et al., unfortunately, no further studies were performed after no significant degradation was observed.^4^ Here we systematically explored whether the PROTACs method can be successfully expanded to E3 ligase DCAF15 with untapped potential in vitro and in vivo.

To design PROTACs hijacking the E3 ligase complex CRL4-DCAF15, BRD4 was selected as the target protein since which is a well-studied member of BET (Bromodomain and extra-terminal domain) family and plays important roles in hematologic and solid tumorigenesis, in addition, it has already been successfully targeted by PROTACs derived from either VHL or CRBN as well (Fig. 1a).^1^ In the previous report, biotinylated photosensitive sulfonamide E7820 probe has been shown less binding affinity loss with DCAF15, which makes the introduction of a linker from the phenyl ring of E7820 is a reasonable choice (Fig. 1a). Twelve compounds were prepared by tethering the BET inhibitor JQ1 to the different positions on the phenyl ring of E7820 with various PEG-linkers lengths. A lymphoma cell line SU-DHL-4 was chosen to evaluate the degradation efficacies of BRD4 by PROTACs. All synthetic PROTACs can degrade BRD4 at micromolar level and the maximum degradation efficacy is above 70% (Supplementary Fig. 1 and Supplementary Table 1). Among them, DP1 was the most potent degrader (DC_50_ = 10.84 ± 0.92 μM, D_max_ = 98%) for further characterization. Treatment of SU-DHL-4 cells with increasing concentrations of DP1, dose-dependent degradation was observed as well as BRD2/3 (Fig. 1b). We then tested DP1 in a panel of other cell lines derived from hematopoietic and lymphoid lineages. We found that DP1 was consistently active in majority cell lines stating at 1 μM (Supplementary Fig. 2 and Supplementary Table 2), which suggested that the extent of substrate depletion is greater though DP1 induced cellular degradation of BRD4 is less potent. Degradation kinetics assay showed an evident reduction of BRD4 protein was observed at 12 hours (Fig. 1c) and the level of BRD4 was not restored even after 72 h upon DP1 washout, suggesting that DP1 is stable in cells and DP1 induced degradation is relatively persistent (Supplementary Fig. 3). Moreover, transcription levels of BRD4 were not downregulated in SU-DHL-4 cells when treated with dosed concentrations of DP1, except c-MYC whose expression is under the control of BET proteins (Supplementary Fig. 4). BRD4 is a well-known nuclear protein, while DCAF15 was less characterized. By constructing a haemagglutinin (HA)-tagged DCAF15, we found the nuclear localization of DCAF15 in transfected 293 T cells (Supplementary Fig. 5), same as DCAF16. These results demonstrated that DP1 mediates the durable nuclear degradation of BRD4.

**Fig. 1:**
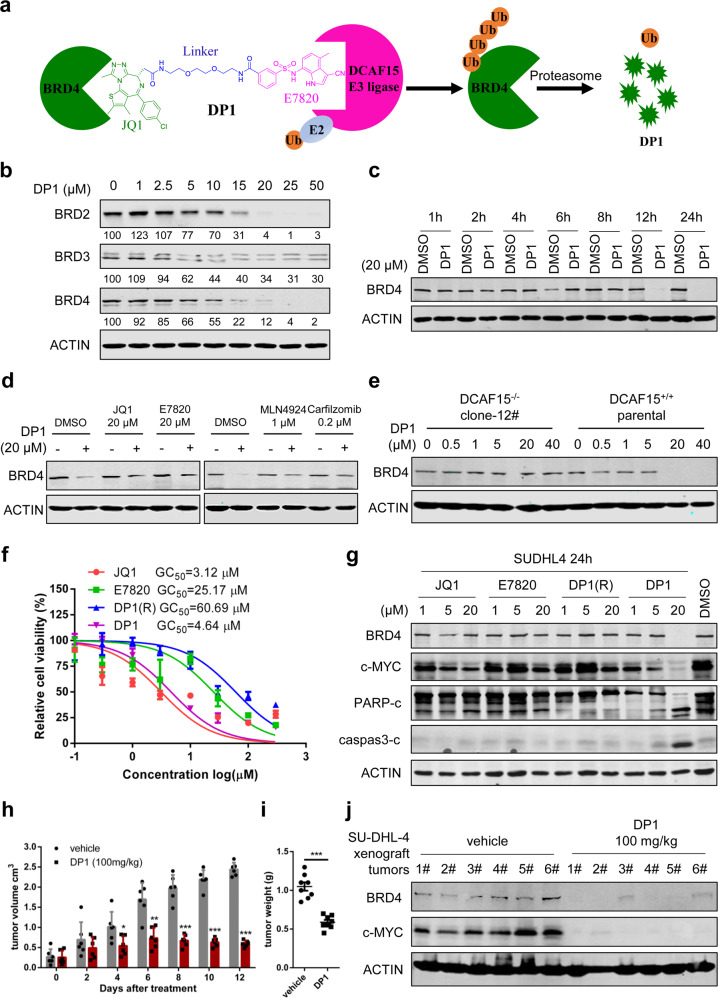
Design and characterization of DCAF15 E3 ligase derived PROTAC to degrade BRD4 in vitro and in vivo. **a** PROTACs designing schematic. **b** Immunoblot of BET protein and ACTIN after 16 h of treatment of SU-DHL-4 cells with the indicated concentrations of DP1. Degradation activity is calculated below each lane as % of protein level relative to DMSO control. **c** Immunoblot of BRD4 and ACTIN after treatment of SU-DHL-4 cells with 20 μM DP1 for the indicated incubation times. **d** Left panel: Immunoblot of BRD4 and ACTIN after a 4 h pretreatment with 20 μM of ligands JQ1 and E7820, followed with a 14 h 20 μM DP1 treatment in SU-DHL-4 cells. Right panel: Immunoblot of BRD4 and ACTIN after a 4 h pretreatment with carfilzomib (0.2 μM) or MLN4924 (1 μM), followed with a 14 h 20 μM DP1 treatment in SU-DHL-4 cells. **e** Immunoblot of BRD4 and ACTIN following treatment of clone 12 and parental cells for 24 h with the indicated concentrations of DP1. **f** Cell viability analysis of SU-DHL-4 cells treated with DP1 for 48 h compared with its component ligands JQ1, E7820, and DP1(R) (*n* = 3). **g** Immunoblot analysis of BRD4, c-MYC, cleaved PARP and Caspase 3, and ACTIN in SU-DHL-4 cells treated with the indicated concentrations of JQ1, E7820, DP1(R), and DP1 for 24 h. **h** Tumor volume of vehicle-treated mice or mice treated with DP1 (100 mg/kg) for 12 days, *n* = 8. **i** Tumor weight of mice treated with vehicle and DP1 after sacrificed. **j** Immunoblot of BRD4, c-MYC, and ACTIN using tumor lysates from mice treated with vehicle and DP1

To investigate the mechanism of DP1-induced BRD4 degradation, we explored whether DP1 induced degradation of BRD4 is dependent on binding to both BRD4 and DCAF15 and proteasome functions. The protein level of BRD4 was not altered after incubating SU-DHL-4 cells with different doses of JQ1 and E7820 for 24 h, which indicated that BRD4 degradation was depended on PROTAC molecules rather than individual ligand itself (Supplementary Fig. 6a). Pre-treatment with equimolar amounts of JQ1 or E7820 inhibited DP1-induced degradation (Fig. 1d, left panel). Requisite engagement with target protein was also confirmed by DP1(R), an inactive PROTAC derivative that contains a stereo-isomer form of JQ1 to deplete its BRD4 binding, failed to elicit BRD4 degradation (Supplementary Fig. 6b). Additionally, both pre-treatment of proteasome inhibitor carfilzomib and the neddylation inhibitor MLN4924 rescued BRD4 degradation (Fig. 1d, right panel). Moreover, DP1 did not degrade BRD4 in DCAF15 knockout SU-DHL-4 cells which further definitively clarified an essential role of DCAF15 in BRD4 degradation (Fig. 1e). Collectively, these data indicated that the degradation of BRD4 relied on ligand-binding, proteasome and an active DCAF15 E3 ligase complex.

We next evaluated the cellular effect of DP1, compared with BET inhibitor JQ1, DCAF15 ligand E7820, and DP1(R). DP1 inhibited cell proliferation comparable with the original ligands of BRD4 in SU-DHL-4 cells in MTS assays (Fig. 1f). DP1 also effectively reduced c-MYC expression and induced apoptosis which was revealed by cleaved PARP and Caspase 3 after 24 h treatment (Fig. 1g) and further quantitatively confirmed by FACS (Supplementary Fig. 7). To validate the potential therapeutic effect of DP1 in vivo, SCID mice bearing the SU-DHL-4 tumors were treated with DP1 (100 mg/kg body weight daily, i.p.) or vehicle control. After 12 days of therapy, administration of DP1 significantly attenuated tumor growth, as evidenced by continuous volumetric measurement (Fig. 1h) and reduced tumor weight evaluated after euthanasia (Fig. 1i). Reduced BRD4 and c-MYC were also observed by immunoblot and immunohistochemistry in the excised xenograft tumors (Fig. 1j and Supplementary Fig. 8), demonstrating that DCAF15 derived PROTAC can be absorbed and distribute into tissues and retain its degradation activity in vivo, though the cellular degradation efficacy is at micromole concentrations. Notably, 12 days administration of DP1 does not induce significant weight loss in the SCID mice (Supplementary Fig. 9).

In summary, we have reported a novel BRD4 degrader DP1 based on E7820 via recruiting the E3 ligase DCAF15, which can induce durable degradation of target proteins and exhibit therapeutic potential in hematologic malignancies both in vitro and in vivo. Although much effort was made to optimize our compounds, the micromolar potencies and flat SAR of these DCAF15-based PROTACs may attribute to these reasons: (1) the limitation of flat binding pocket of DCAF15 which had been proved recently.^5^ (2) Different from the high affinities of IMiDs to CRBN, sulfonamides were bound weakly with DCAF15.^2^ Besides our report, Zoppi et al. had designed two indisulam-based PROTACs targeted BRD7/9 via recruiting DCAF15 E3 ligase but without observing significant degradations,^4^ which may due to the different accessibility to form ternary complex (targets-PROTAC-DCAF15) and different cell lines used in experiments. Therefore, the development of DCAF15 ligands with higher affinity and specificity based on reported structural information of ternary complex composed of E7820, RBM39 and DCAF15 E3 ligase^5^ would be valuable for guiding DCAF15-based PROTACs design in the future. Collectively, our study provides a series of evidence that the DCAF15 can serve as a valuable addition to the limited E3 ligases used in PROTACs design.

## Supplementary information

Supplementary Materials

Supplementary Information
