# Supplementary material for: In vivo target protein degradation induced by PROTACs based on E3 ligase DCAF15
Source: Signal Transduct Target Ther. 2020 Jul 27;5:129. doi: 10.1038/s41392-020-00245-0 (PMC7383041; doi:10.1038/s41392-020-00245-0)
Supplement: Supplementary file 1 — Supplementary Materials [file 41392_2020_245_MOESM1_ESM.docx]

Supplementary Materials for

*In vivo* target protein degradation induced by PROTACs based on E3 ligase DCAF15

Liang Li^1,2^, Dazhao Mi^1^, Haixiang Pei^1^, Qiuhui Duan^1^, Xinyue Wang^2^, Wenbo Zhou^1^, Jianping Jin^3^, Dali Li^1^, Mingyao Liu^1^ and Yihua Chen^1^

^1^ Shanghai Key Laboratory of Regulatory Biology, Joint Research Center for Translational Medicine, ECNU-Fengxian Hospital, Institute of Biomedical Sciences and School of Life Sciences, East China Normal University, Shanghai 200241, China

^2^ Joint Center for Translational Medicine, Fengxian District Central Hospital, Shanghai 201499, China

^3^ MOE Key Laboratory for Biosystems Homeostasis & Protection and Innovation Center for Cell Signaling Network, Life Sciences Institute, Zhejiang University, Hangzhou, Zhejiang 310058, China

These authors contributed equally: Liang Li, Dazhao Mi and Haixiang Pei

Correspondence: [yhchen@bio.ecnu.edu.cn](mailto:yhchen@bio.ecnu.edu.cn) (Y. Chen) or [myliu@bio.ecnu.edu.cn](mailto:myliu@bio.ecnu.edu.cn) (M. Liu)

**This file includes:**

Materials and Methods

Supplementary Text

Figures. S1 to S9

Tables. S1 to S3

**Materials and Methods**

Compounds and chemical syntheses

MLN4924 (HY-70062), Carfilzomib (HY-10455), E7820 (HY-14571) were all purchased from MedChem Express. See the Supplementary Text - Chemical part for synthetic schemes and validations of other compounds and PROTACs.

Cell lines

Toledo (RPMI 1640 supplemented with 10% FBS), Farage (RPMI 1640 supplemented with 10% FBS), MV-4-11 (IMDM supplemented with 10% FBS (Gibco, Thermo)), Daudi (RPMI 1640 supplemented with 10% FBS), Raji (RPMI 1640 supplemented with 10% FBS), SU-DHL-4 (RPMI 1640 supplemented with 10% FBS), Jurkat (RPMI 1640 supplemented with 10% FBS), K562 (IMDM supplemented with 10% FBS) and normal 293T cell line (DMEM supplemented with 10% FBS) were obtained from the American Type Culture Collection (ATCC). OCI-LY7 (IMDM supplemented with 10% FBS) was purchased from the German collection of microorganisms and cell cultures (DSMZ). All cells were cultured at 37°C with 5% CO2 and supplemented with 100 U/mL penicillin, 100 μg/mL streptomycin (Gibco). All cell lines were used in experiments for no longer than 20 passages in total. Mycoplasma testing of all cultured cell lines was performed every four weeks using the MycoAlert detection kit (Invitrogen, M7006).

Immunoblotting

Cells were collected at 200 g for 5 min and lysed for 15 min on ice using RIPA buffer supplemented with protease inhibitor cocktail (MedChem Express, HY-K0010). The lysates were spun at 12,000 rpm for 15 min at 4°C and protein concentration was performed using the BCA Protein assay kit (Thermo, 23225). The following primary antibodies used in this study: BRD4 (Abcam, ab128874), Actin (Sigma), c-MYC (Cell Signaling Technology, #13987), PARP (Cell Signaling Technology, #9532) and Cleaved Caspase-3 (Cell Signaling Technology, #5848). Fluorescence-labeled secondary antibodies (Sigma) and the OdysseyCLxImager (LI-COR) were used to image and visualize the immunoblot. Quantification of band intensities was performed using Image J software and graphed using Prism V6.0 (GraphPad Software).

Cell viability assay

Cells were counted and seeded into 96-well plates and incubated with the indicated concentrations of the PROTACs or component ligands for 48 hours, and anti-proliferative effects were assessed using the CellTiter-Glo kit (Promega). Viable cells were quantitated by measuring absorbance at 490 nm using a SPECTROstar^Nano^ (BMG LABTECH). Data were analyzed and graphed with GraphPad Prism (GraphPad Software) nonlinear regression curve fit.

Lentivirus production and generation of DCAF15-deficient cell line

Lentivirus production was conducted in 293T cells by co-transfection of pMD2G, psPAX2 (Addgene #12259 and #12260, respectively), and a lentiCRISPRv2 plasmid containing a sgRNA targeting the exon 1 of the DCAF15 gene (GAAATCGgagcggaacagcg) using PEI (Polysciences, 24765-2). Viral supernatants were collected 48 and 72 hours after transfection, filtered through a 0.4 μm membrane and concentrated via ultracentrifugation (Beckman Coulter). SU-DHL-4 cells were then transduced by incubation with concentrated viral particles and 8 μg/mL polybrene (Sigma). After 48 h, transduced cells were screened using 2 μg/mL puro for five days. Single-cell clones were seeded, expanded and analyzed for DCAF15 knockout by sequencing for genome modification at the DCAF15 locus.

RNA isolation and analysis

Cells of indicated samples were washed with PBS and pelleted to remove excess PBS before the addition of TRIzol for RNA extraction. Total RNA was extracted using the TRIzol reagent (Takara). cDNAs were synthesized using the PrimeScript RT Master Mix (Takara). Subsequent PCRs were performed using the Power SYBR Green PCR Master kit (Takara) on the QuantStudio 3 Real-Time PCR System (Applied Biosystems) using the comparative Ct quantization method. Results were normalized to ACTIN *mRNA* levels. All Primer sequences are listed in Table S3.

Xenograft experiment (subcutaneous)

Animal experiments were conducted in the Animal Research Center of East China Normal University in accordance with guidelines and regulations drafted by the Association for Assessment and Accreditation of Laboratory Animal Care in Shanghai (Ethical number: m20190704). Xenograft tumors were established by injecting 1 × 10^7^ SU-DHL-4 cells suspended in 50% Matrigel (Corning, 356237) subcutaneously on the dorsal side of severe combined immunodeficient (SCID) mice, one tumor per mouse. When tumor volume reached about 100 mm^3^, mice were randomly divided into the control (vehicle) or the DP1 treated groups. Mice were treated with 100 mg/kg DP1 or vehicle via intraperitoneal injection every day. Tumor size was measured utilizing electronic calipers every other day during the treatment period. Tumor volume was calculated as *V = L × W^2^ / 2*, where L is the length and W is the width of the xenograft tumor.

Immunofluorescence (IF)

Transfected cells were seeded and grown on a 15mm glass slide placed in a 24 well plate. After 24 hours, cells were rinsed three times with cold PBS and fixed with 4% paraformaldehyde for 15 min at room temperature. The fixed cells were rinsed, permeabilized and blocked with 5% BSA for 30 min at room temperature. Then the cells were incubated overnight at 4°C with HA antibody (SANTA CRUZ, sc-805) at 1:100 dilution. After incubating with Alexa Fluor 488-conjugated goat anti-rabbit IgG secondary antibody and counterstained with DAPI, cells were imaged Leica microscope (Leica, DM4000b) and processed in Image J software.

Statistical analysis

Statistical analysis was performed via GraphPad Prism 6.0 software. All values are presented as the mean ± SD. The student t-test was used in analyzing the statistical significance of experiments comparing two groups. For multiple comparisons, one-way or two-way ANOVA was used. All immunoblotting experiments and cell viability and apoptosis assay were done at least in triplicate per condition or repeated for three times. Differences were considered statistically significant at *p* < 0.05 unless otherwise indicated.

**Supplementary Text**

Chemistry - Chemical synthesis and structure validation

All reagents were purchased from Bidepharm, Aladdin-Reagents Inc and were used without purification. The ^1^H NMR spectra were measured on a Bruker 500 MHz instrument in DMSO-*d*_6_ (recorded in ppm). DMSO-*d*_6_ (2.50 ppm) was used as the internal reference, Coupling constants (*J*) are reported in hertz (Hz). High-resolution mass spectra (HRMS) were measured on a Bruker MicroTOF-Q II liquid chromatography (LC)–mass spectrometry (MS) instrument operating in electrospray ionization (ESI). High-performance liquid chromatography (HPLC) (Agilent Technologies 1200 Series) was carried out for purity determination using the Eclipse XDB C18 column (5 μm, 4.6 mm × 150 mm) at 40 °C for column temperature by a mixture of solvent water/methanol, the UV detection wavelength is 254 nm; flow rate = 1.5 mL/min; solvent A: water; solvent B: MeOH; gradient of 40-90% B (0-10 min), 90% B (10-15 min), 90-40% B (15-20 min).

**The common synthetic route of compound 1 ~ 12**

**Compound 1, 3, 5, 7, 9, 11:** Linkers are located in the para of the benzene ring of E7820, **Compound 2, 4, 6, 8, 10, 12:** Linkers are located in the meta of the benzene ring of E7820.

**The synthetic method of (*S*)-*N*-(2-(2-(2-(2-(4-(4-chlorophenyl)-2,3,9-trimethyl-6*H*-thieno[3,2-*f*][1,2,4]triazolo[4,3-*a*][1,4]diazepin-6-yl)acetamido)ethoxy)ethoxy)ethyl)-3-(*N*-(3-cyano-4-methyl-1*H*-7-indolyl)sulfamoyl)benzamide (DP1/Compound 4).**

To a mixture of 7-amino-4-methyl-1*H*-indole-3-carbonitrile (500 mg, 2.93 mmol) and pyridine (530 mg, 7.33 mmol) in 20 mL of THF at 0 ℃ was slowly added the solution of 3-(chlorosulfonyl) benzoic acid (970 mg, 4.40 mmol, dissolved in 10 mL of THF), then the reaction mixture was stirred at room temperature overnight. The resulting mixture was quenched by saturated ammonium chloride and extracted by EtOAc, the organic phase was dried over anhydrous Na_2_SO_4_ and evaporated in vacuum, the crude product was purified by column chromatography (DCM/MeOH = 50/1) to get the 3-(*N*-(3-cyano-4-methyl-1*H*-7-indolyl)sulfamoyl)benzoic acid (750 mg, 72% yield).

A mixture of 3-(*N*-(3-cyano-4-methyl-1*H*-7-indolyl)sulfamoyl)benzoic acid (50 mg, 0.15 mmol), HATU (74 mg, 0.19 mmol), *tert*-butyl (2-(2-(2-aminoethoxy)ethoxy)-ethyl)carbamate (41 mg, 0.17 mmol) and DIEA (54 mg, 0.42 mmol) in 2 mL of DMF was stirred at room temperature for 5 h. Then the reaction mixture was extracted by EtOAc and washed twice with saturated aqueous NaCl, the organic phase was dried over anhydrous Na_2_SO_4_ and evaporated in vacuum. The crude product was purified by column chromatography (DCM/MeOH = 80/1) to get the *tert*-butyl (2-(2-(2-(3-(*N*- (3-cyano-4-methyl-1*H*-7-indolyl)sulfamoyl)benzamido)ethoxy)ethoxy)ethyl)carbamate (57 mg, 69% yield).

The intermediate *tert*-butyl (2-(2-(2-(3-(*N*-(3-cyano-4-methyl-1*H*-7-indolyl) sulfamoyl)benzamido) -ethoxy)ethoxy)ethyl)carbamate was dissolved in 2 mL of DCM which was added three drops of TFA. The mixture was stirred at room temperature for 3 h to get the product which was used the next step directly.

A mixture of (*S*)-2-(4-(4-chlorophenyl)-2,3,9-trimethyl-6*H*-thieno[3,2-*f*][1,2,4] triazolo[4,3-*a*]-[1,4]diazepin-6-yl)acetic acid (40 mg, 0.10 mmol), HATU (50 mg, 0.13 mmol), DIEA (39 mg, 0.30 mmol) and the intermediate from previous step in 2 mL of DMF was stirred at room temperature for 6 h. Then the reaction mixture was extracted by EtOAc and washed twice with saturated aqueous NaCl, the organic phase was dried over anhydrous Na_2_SO_4_ and evaporated in vacuum. The crude product was purified by column chromatography (DCM/MeOH = 100/1) to get 67 mg (77% yield) (*S*)-*N*-(2-(2-(2-(2-(4-(4-chlorophenyl)-2,3,9-trimethyl-6*H*-thieno[3,2-*f*][1,2,4]triazolo[4,3-*a*]-[1,4]diazepin-6-yl)acetamido) ethoxy)ethoxy)ethyl)-3-(*N*-(3-cyano-4-methyl-1*H*-7-indolyl)-sulfamoyl)benzamide **(DP1/Compound 4).** ^1^H NMR (500 MHz, DMSO-*d*_6_) δ 11.99 (d, *J* = 3.2 Hz, 1H), 10.02 (s, 1H), 8.77 (t, *J* = 5.6 Hz, 1H), 8.28 (t, *J* = 5.7 Hz, 1H), 8.24 (s, 1H), 8.17 (d, *J* = 3.0 Hz, 1H), 8.07 (d, *J* = 7.7 Hz, 1H), 7.78 (d, *J* = 7.9 Hz, 1H), 7.65 – 7.59 (m, 1H), 7.48 (d, *J* = 8.4 Hz, 2H), 7.42 (d, *J* = 8.4 Hz, 2H), 6.76 (d, *J* = 7.8 Hz, 1H), 6.52 (d, *J* = 7.7 Hz, 1H), 4.51 (dd, *J* = 7.9, 6.2 Hz, 1H), 3.60 – 3.39 (m, 10H), 3.31 – 3.18 (m, 4H), 2.59 (s, 3H), 2.56 (s, 3H), 2.40 (s, 3H), 1.61 (s, 3H); ^13^C NMR (150 MHz, Chloroform-*d*) δ 171.36, 165.99, 165.08, 155.78, 150.84, 139.50, 137.15, 136.34, 135.40, 133.17, 132.74, 132.07, 131.62, 131.04, 130.65, 130.38, 130.02, 130.00, 129.31, 128.93, 128.82, 126.96, 124.70, 123.17, 119.87, 119.59, 117.42, 86.56, 70.44, 69.98, 69.78, 69.64, 54.33, 40.15, 39.70, 39.00, 18.31, 14.48, 13.26, 11.87; HPLC purity: 98.8%, R_t_ = 9.10 min; HRMS (ESI): calcd for C_42_H_43_ClN_9_O_6_S_2_ [M+H]^+^: 868.2461, found 868.2472.

**Following the same synthetic methods to get other compounds (Compound 1, 2, 3, 5, 6, 7, 8, 9, 10, 11, 12), except using different types and lengths mono-Boc protected diamines.**

1. **-*N*-(2-(2-(2-(4-(4-chlorophenyl)-2,3,9-trimethyl-6*H*-thieno[3,2-*f*][1,2,4]triazolo[4,3-*a*] [1,4]diazepin-6-yl)acetamido)ethoxy)ethyl)-4-(*N*-(3-cyano-4-methyl-1*H*-7-indolyl)sulfamoyl)-benzamide (Compound 1)**. ^1^H NMR (500 MHz, DMSO-*d*_6_) δ 11.99 (d, *J* = 3.2 Hz, 1H), 10.04 (s, 1H), 8.70 (t, *J* = 5.5 Hz, 1H), 8.29 (t, *J* = 5.6 Hz, 1H), 8.18 (d, *J* = 3.0 Hz, 1H), 7.95 (d, *J* = 8.5 Hz, 2H), 7.74 (d, *J* = 8.5 Hz, 2H), 7.46 (d, *J* = 8.8 Hz, 2H), 7.41 (d, *J* = 8.5 Hz, 2H), 6.76 (d, *J* = 8.3 Hz, 1H), 6.52 (d, *J* = 7.7 Hz, 1H), 4.51 (dd, *J* = 8.2, 6.0 Hz, 1H), 3.57 (t, *J* = 6.0 Hz, 2H), 3.54 – 3.42 (m, 4H), 3.33 – 3.11 (m, 4H), 2.59 (s, 3H), 2.55 (s, 3H), 2.40 (s, 3H), 1.60 (s, 3H); ^13^C NMR (125 MHz, Chloroform-*d*) δ 171.67, 166.01, 165.70, 155.63, 150.52, 141.71, 138.35, 137.20, 136.40, 133.68, 132.84, 131.60, 131.16, 131.03, 130.91, 130.26, 129.98, 129.34, 129.05, 128.93, 127.80, 127.16, 126.97, 123.27, 120.40, 119.98, 117.52, 86.76, 69.52, 69.35, 54.61, 40.18, 40.00, 38.94, 18.27, 14.34, 13.19, 11.84. HPLC purity: 91.5%, R_t_ = 9.94 min; HRMS (ESI): calcd for C_40_H_38_ClN_9_NaO_5_S_2_ [M+Na]^+^: 846.2018, found 846.2000.

**(*S*)-*N*-(2-(2-(2-(4-(4-chlorophenyl)-2,3,9-trimethyl-6*H*-thieno[3,2-*f*][1,2,4]triazolo[4,3-*a*] [1,4]diazepin-6-yl)acetamido)ethoxy)ethyl)-3-(*N*-(3-cyano-4-methyl-1*H*-7-indolyl)sulfamoyl) benzamide (Compound 2).** ^1^H NMR (500 MHz, DMSO-*d*_6_) δ 11.98 (s, 1H), 10.02 (s, 1H), 8.73 (t, *J* = 5.5 Hz, 1H), 8.31 (t, *J* = 5.7 Hz, 1H), 8.26 – 8.23 (m, 1H), 8.17 (s, 1H), 8.09 (d, *J* = 7.8 Hz, 1H), 7.77 (d, *J* = 7.9 Hz, 1H), 7.60 – 7.55 (m, 1H), 7.47 (d, *J* = 8.2 Hz, 2H), 7.41 (d, *J* = 8.2 Hz, 2H), 6.75 (d, *J* = 7.7 Hz, 1H), 6.52 (d, *J* = 7.5 Hz, 1H), 4.51 (dd, *J* = 8.0, 6.1 Hz, 1H), 3.60 – 3.42 (m, 8H), 3.29 – 3.22 (m, 2H), 2.62 (s, 3H), 2.59 (s, 3H), 2.55 (s, 3H), 2.39 (s, 3H), 1.60 (s, 3H);^13^C NMR (125 MHz, Chloroform-*d*) δ 171.65, 165.98, 165.65, 155.64, 150.49, 138.96, 137.18, 136.02, 135.71, 133.18, 132.84, 132.21, 131.58, 131.15, 130.68, 130.50, 130.32, 129.86, 129.63, 128.79, 128.64, 126.97, 124.81, 122.96, 120.26, 119.01, 117.25, 86.76, 69.53, 69.35, 54.62, 40.17, 40.00, 38.96, 18.27, 14.34, 13.19, 11.85. HPLC purity: 95.5%, R_t_ = 8.61 min; HRMS (ESI): calcd for C_40_H_38_ClN_9_NaO_5_S_2_ [M+Na]^+^: 846.2018, found 846.2032.

**(*S*)-*N*-(2-(2-(2-(2-(4-(4-chlorophenyl)-2,3,9-trimethyl-6*H*-thieno[3,2-*f*][1,2,4]triazolo[4,3-*a*] [1,4]diazepin-6-yl)acetamido)ethoxy)ethoxy)ethyl)-4-(*N*-(3-cyano-4-methyl-1*H*-7-indolyl)-sulfamoyl)benzamide (Compound 3).** ^1^H NMR (500 MHz, DMSO-*d*_6_) δ 11.99 (s, 1H), 10.05 (s, 1H), 8.73 (t, *J* = 5.9 Hz, 1H), 8.28 (t, *J* = 6.1 Hz, 1H), 8.18 (s, 1H), 7.94 (d, *J* = 8.4 Hz, 2H), 7.76 (d, *J* = 8.4 Hz, 2H), 7.48 (d, *J* = 8.5 Hz, 2H), 7.42 (d, *J* = 8.7 Hz, 2H), 6.77 (d, *J* = 7.7 Hz, 1H), 6.52 (d, *J* = 7.7 Hz, 1H), 4.51 (dd, *J* = 8.1, 6.0 Hz, 1H), 3.64 – 3.17 (m, 14H), 2.59 (s, 3H), 2.56 (s, 3H), 2.40 (s, 3H), 1.61 (s, 3H); ^13^C NMR (150 MHz, Chloroform-*d*) δ 170.34, 167.91, 166.13, 155.62, 151.29, 141.63, 138.11, 137.49, 136.38, 133.14, 132.58, 132.03, 131.41, 131.03, 130.91, 130.19, 130.03, 129.30, 129.10, 128.94, 127.71, 127.20, 126.97, 123.38, 120.47, 119.77, 117.48, 86.68, 70.67, 70.58, 70.46, 70.07, 54.29, 40.15, 39.58, 38.87, 18.36, 14.61, 13.34, 12.05; HPLC purity: 96.4%, R_t_ = 8.46 min; HRMS (ESI): calcd for C_42_H_42_ClN_9_NaO_6_S_2_ [M+Na]^+^: 890.2280, found 890.2282.

**(*S*)-*N*-(1-(4-(4-chlorophenyl)-2,3,9-trimethyl-6*H*-thieno[3,2-*f*][1,2,4]triazolo[4,3-*a*][1,4]diazepin-6-yl)-2-oxo-6,9,12-trioxa-3-azatetradecan-14-yl)-4-(*N*-(3-cyano-4-methyl-1*H*-7-indolyl)sulfamoyl)- benzamide (Compound 5).** ^1^H NMR (500 MHz, DMSO-*d*_6_) δ 11.99 (s, 1H), 10.04 (s, 1H), 8.71 (t, *J* = 5.6 Hz, 1H), 8.27 (t, *J* = 5.9 Hz, 1H), 8.18 (d, *J* = 3.0 Hz, 1H), 7.94 (d, *J* = 8.1 Hz, 2H), 7.76 (d, *J* = 8.2 Hz, 2H), 7.48 (d, *J* = 8.2 Hz, 2H), 7.42 (d, *J* = 8.2 Hz, 2H), 6.77 (d, *J* = 7.7 Hz, 1H), 6.52 (d, *J* = 7.7 Hz, 1H), 4.51 (m, 1H), 3.57 – 3.49 (m, 10H), 3.47 – 3.38 (m, 4H), 3.31 – 3.18 (m, 4H), 2.59 (s, 3H), 2.56 (s, 3H), 2.40 (s, 3H), 1.62 (s, 3H); ^13^C NMR (125 MHz, Chloroform-*d*) δ 170.97, 166.46, 164.97, 155.71, 150.80, 141.60, 138.37, 137.35, 136.36, 133.31, 131.83, 131.79, 131.24, 131.02, 130.92, 130.23, 129.97, 129.34, 129.02, 128.94, 127.74, 127.15, 126.97, 123.35, 120.55, 119.88, 117.53, 86.59, 70.57, 70.47, 70.21, 70.02, 69.84, 69.74, 54.22, 40.07, 39.61, 38.87, 18.38, 14.54, 13.31, 11.92. HPLC purity: 96.5%, R_t_ = 8.54 min; HRMS (ESI): calcd for C_44_H_46_ClN_9_NaO_7_S_2_ [M+Na]^+^: 934.2542, found 934.2530.

**(*S*)-*N*-(1-(4-(4-chlorophenyl)-2,3,9-trimethyl-6*H*-thieno[3,2-*f*][1,2,4]triazolo[4,3-*a*][1,4]diazepin-6-yl)-2-oxo-6,9,12-trioxa-3-azatetradecan-14-yl)-3-(*N*-(3-cyano-4-methyl-1*H*-7-indolyl)sulfamoyl)-benzamide (Compound 6).** ^1^H NMR (500 MHz, DMSO-*d*_6_) δ 11.99 (d, *J* = 3.1 Hz, 1H), 10.02 (s, 1H), 8.76 (t, *J* = 5.6 Hz, 1H), 8.27 (t, *J* = 5.7 Hz, 1H), 8.24 (s, 1H), 8.18 (d, *J* = 3.1 Hz, 1H), 8.07 (d, *J* = 7.9 Hz, 1H), 7.79 (d, *J* = 7.7 Hz, 1H), 7.65 – 7.58 (m, 1H), 7.48 (d, *J* = 8.7 Hz, 2H), 7.42 (d, *J* = 8.5 Hz, 2H), 6.76 (d, *J* = 8.4 Hz, 1H), 6.52 (d, *J* = 7.7 Hz, 1H), 4.51 (dd, *J* = 8.0, 6.1 Hz, 1H), 3.57 – 3.18 (m, 18H), 2.59 (s, 3H), 2.56 (s, 3H), 2.40 (s, 3H), 1.62 (s, 3H); ^13^C NMR (125 MHz, Chloroform-*d*) δ 171.52, 165.85, 164.99, 156.08, 151.19, 139.56, 137.09, 136.56, 135.33, 133.17, 132.86, 132.39, 131.59, 131.02, 130.86, 130.54, 130.04, 129.81, 129.73, 128.98, 128.85, 126.99, 124.76, 123.21, 120.03, 118.85, 117.49, 86.61, 70.94, 70.51, 70.43, 70.19, 70.08, 69.48, 54.18, 40.43, 39.83, 38.79, 18.31, 14.51, 13.31, 11.94. HPLC purity: 97.4%, R_t_ = 8.63 min; HRMS (ESI): calcd for C_44_H_46_ClN_9_NaO_7_S_2_ [M+Na]^+^: 934.2542, found 934.2548.

**(*S*)-*N*-(1-(4-(4-chlorophenyl)-2,3,9-trimethyl-6*H*-thieno[3,2-*f*][1,2,4]triazolo[4,3-*a*][1,4]diazepin-6-yl)-2-oxo-6,9,12,15-tetraoxa-3-azaheptadecan-17-yl)-4-(*N*-(3-cyano-4-methyl-1*H*-7-indolyl)- sulfamoyl)benzamide (Compound 7)**. ^1^H NMR (500 MHz, DMSO-*d*_6_) δ 11.98 (s, 1H), 10.04 (s, 1H), 8.71 (t, *J* = 5.7 Hz, 1H), 8.27 (t, *J* = 5.8 Hz, 1H), 8.17 (s, 1H), 7.93 (d, *J* = 8.2 Hz, 2H), 7.76 (d, *J* = 8.1 Hz, 2H), 7.48 (d, *J* = 8.3 Hz, 2H), 7.42 (d, *J* = 8.3 Hz, 2H), 6.76 (d, *J* = 7.8 Hz, 1H), 6.52 (d, *J* = 7.7 Hz, 1H), 4.51 (dd, *J* = 8.0, 6.0 Hz, 1H), 3.57 – 3.19 (m, 22H), 2.59 (s, 3H), 2.56 (s, 3H), 2.40 (s, 3H), 1.62 (s, 3H); ^13^C NMR (125 MHz, Chloroform-*d*) δ 171.34, 166.51, 165.01, 155.75, 150.69, 141.87, 138.62, 137.33, 136.67, 133.73, 132.70, 132.63, 131.47, 131.02, 130.77, 130.47, 130.11, 129.85, 129.50, 128.93, 128.77, 126.68, 124.96, 123.18, 119.97, 119.32, 117.70, 86.32, 70.76, 70.65, 70.55, 70.45, 70.36, 70.26, 70.21, 69.98, 54.14, 40.24, 39.73, 38.87, 18.33, 14.27, 13.32, 12.00. HPLC purity: 97.5%, R_t_ = 8.62 min; HRMS (ESI): calcd for C_46_H_50_ClN_9_NaO_8_S_2_ [M+Na]^+^: 978.2804, found 978. 2798.

**(*S*)-*N*-(1-(4-(4-chlorophenyl)-2,3,9-trimethyl-6*H*-thieno[3,2-*f*][1,2,4]triazolo[4,3-*a*][1,4]diazepin-6-yl)-2-oxo-6,9,12,15-tetraoxa-3-azaheptadecan-17-yl)-3-(*N*-(3-cyano-4-methyl-1*H*-7-indolyl)-sulfamoyl)benzamide (Compound 8).** ^1^H NMR (500 MHz, DMSO-*d*_6_) δ 11.98 (d, *J* = 3.2 Hz, 1H), 10.02 (s, 1H), 8.76 (t, *J* = 5.6 Hz, 1H), 8.27 (t, *J* = 5.7 Hz, 1H), 8.24 (s, 1H), 8.17 (d, *J* = 3.0 Hz, 1H), 8.07 (d, *J* = 7.8 Hz, 1H), 7.79 (d, *J* = 8.0 Hz, 1H), 7.64 – 7.59 (m, 1H), 7.48 (d, *J* = 8.5 Hz, 2H), 7.42 (d, *J* = 8.5 Hz, 2H), 6.76 (d, *J* = 7.7 Hz, 1H), 6.52 (d, *J* = 7.7 Hz, 1H), 4.51 (dd, *J* = 8.1, 6.0 Hz, 1H), 3.59 – 3.47 (m, 14H), 3.47 – 3.38 (m, 4H), 3.33 – 3.18 (m, 4H), 2.59 (s, 3H), 2.56 (s, 3H), 2.40 (s, 3H), 1.62 (s, 3H); ^13^C NMR (150 MHz, Chloroform-*d*) δ 171.23, 166.73, 164.99, 155.99, 151.01, 139.58, 137.08, 136.50, 135.32, 133.59, 132.43, 132.22, 131.53, 131.00, 130.63, 130.19, 130.12, 129.77, 129.15, 128.92, 128.79, 126.92, 125.15, 123.18, 120.03, 119.72, 117.60, 86.30, 70.74, 70.60, 70.36, 70.20, 69.86, 69.82, 54.09, 40.05, 39.43, 38.65, 18.32, 14.51, 13.28, 11.91; HPLC purity: 98.8%, R_t_ = 8.71 min; HRMS (ESI): calcd for C_46_H_50_ClN_9_NaO_8_S_2_ [M+Na]^+^: 978.2804, found 978.2814.

**(*S*)-*N*-(1-(4-(4-chlorophenyl)-2,3,9-trimethyl-6*H*-thieno[3,2-*f*][1,2,4]triazolo[4,3-*a*][1,4]diazepin-6-yl)-2-oxo-6,9,12,15,18-pentaoxa-3-azaicosan-20-yl)-4-(*N*-(3-cyano-4-methyl-1*H*-7-indolyl)-sulfamoyl)benzamide (Compound 9).** ^1^H NMR (500 MHz, DMSO-*d*_6_) δ 11.98 (d, *J* = 3.2 Hz, 1H), 10.04 (s, 1H), 8.70 (t, *J* = 5.6 Hz, 1H), 8.27 (t, *J* = 5.7 Hz, 1H), 8.18 (d, *J* = 2.9 Hz, 1H), 7.94 (d, *J* = 8.2 Hz, 2H), 7.76 (d, *J* = 8.1 Hz, 2H), 7.48 (d, *J* = 8.3 Hz, 2H), 7.42 (d, *J* = 8.3 Hz, 2H), 6.77 (d, *J* = 7.7 Hz, 1H), 6.52 (d, *J* = 7.7 Hz, 1H), 4.51 (dd, *J* = 8.0, 6.0 Hz, 1H), 3.56 – 3.37 (m, 24H), 3.30 – 3.21 (m, 2H), 2.59 (s, 3H), 2.56 (s, 3H), 2.40 (s, 3H), 1.62 (s, 3H); ^13^C NMR (150 MHz, Chloroform-*d*) δ 171.32, 166.93, 164.60, 155.74, 150.66, 141.74, 138.48, 137.20, 136.43, 133.68, 132.58, 131.77, 131.73, 131.20, 131.02, 130.90, 130.32, 130.05, 129.32, 128.93, 127.99, 127.23, 127.00, 123.28, 120.51, 119.75, 117.59, 86.36, 70.29, 70.22, 70.10, 70.05, 70.02, 69.98, 69.93, 54.25, 40.11, 39.55, 38.61, 18.36, 14.54, 13.29, 11.89; HPLC purity: 96.7%, R_t_ = 8.64 min; HRMS (ESI): calcd for C_48_H_54_ClN_9_NaO_9_S_2_ [M+Na]^+^: 1022.3067, found 1022.3048.

**(*S*)-*N*-(1-(4-(4-chlorophenyl)-2,3,9-trimethyl-6*H*-thieno[3,2-*f*][1,2,4]triazolo[4,3-*a*][1,4]diazepin-6-yl)-2-oxo-6,9,12,15,18-pentaoxa-3-azaicosan-20-yl)-3-(*N*-(3-cyano-4-methyl-1*H*-7-indolyl)- sulfamoyl)benzamide (Compound 10)**. ^1^H NMR (500 MHz, DMSO-*d*_6_) δ 11.98 (d, *J* = 3.1 Hz, 1H), 10.02 (s, 1H), 8.76 (t, *J* = 5.6 Hz, 1H), 8.28 (t, *J* = 5.7 Hz, 1H), 8.24 (s, 1H), 8.17 (d, *J* = 2.8 Hz, 1H), 8.07 (d, *J* = 7.9 Hz, 1H), 7.79 (d, *J* = 8.1 Hz, 1H), 7.65 – 7.59 (m, 1H), 7.48 (d, *J* = 8.7 Hz, 2H), 7.42 (d, *J* = 8.5 Hz, 2H), 6.76 (d, *J* = 8.3 Hz, 1H), 6.52 (d, *J* = 7.7 Hz, 1H), 4.51 (dd, *J* = 8.1, 6.0 Hz, 1H), 3.57 – 3.17 (m, 26H), 2.59 (s, 3H), 2.56 (s, 3H), 2.40 (s, 3H), 1.62 (s, 3H); ^13^C NMR (150 MHz, Chloroform-*d*) δ 171.57, 166.46, 164.71, 155.94, 150.76, 139.59, 137.08, 136.54, 135.36, 133.51, 132.46, 132.16, 131.53, 131.02, 130.64, 130.54, 130.07, 129.89, 129.35, 128.93, 128.82, 126.96, 125.37, 123.19, 120.28, 119.70, 117.55, 86.37, 70.27, 70.21, 70.10, 70.06, 70.01, 69.98, 69.96, 69.78, 54.18, 40.16, 39.50, 38.51, 18.35, 14.52, 13.28, 11.92; HPLC purity: 97.4%, R_t_ = 8.74 min; HRMS (ESI): calcd for C_48_H_54_ClN_9_NaO_9_S_2_ [M+Na]^+^: 1022.3067, found 1022.3070.

**(*S*)-*N*-(1-(4-(4-chlorophenyl)-2,3,9-trimethyl-6*H*-thieno[3,2-*f*][1,2,4]triazolo[4,3-*a*][1,4]diazepin-6-yl)-2-oxo-6,9,12,15,18,21-hexaoxa-3-azatricosan-23-yl)-4-(*N*-(3-cyano-4-methyl-1*H*-7-indolyl)-sulfamoyl)benzamide (Compound 11)**. ^1^H NMR (500 MHz, DMSO-*d*_6_) δ 11.98 (d, *J* = 3.3 Hz, 1H), 10.04 (s, 1H), 8.71 (t, *J* = 5.6 Hz, 1H), 8.28 (t, *J* = 5.7 Hz, 1H), 8.18 (d, *J* = 2.9 Hz, 1H), 7.94 (d, *J* = 8.4 Hz, 2H), 7.77 (d, *J* = 8.3 Hz, 2H), 7.49 (d, *J* = 8.4 Hz, 2H), 7.43 (d, *J* = 8.3 Hz, 2H), 6.77 (d, *J* = 7.7 Hz, 1H), 6.53 (d, *J* = 7.6 Hz, 1H), 4.51 (dd, *J* = 8.1, 6.0 Hz, 1H), 3.63 – 3.16 (m, 30H), 2.59 (s, 3H), 2.56 (s, 3H), 2.41 (s, 3H), 1.62 (s, 3H); ^13^C NMR (150 MHz, Chloroform-*d*) δ 171.26, 167.01, 164.61, 155.73, 150.63, 141.79, 138.30, 137.13, 136.47, 133.83, 132.57, 131.81, 131.68, 131.17, 131.02, 130.85, 130.30, 130.05, 129.23, 128.93, 128.90, 128.00, 126.98, 123.24, 120.40, 119.74, 117.62, 86.23, 70.31, 70.05, 70.02, 69.80, 69.74, 69.71, 54.24, 40.03, 39.47, 38.55, 18.34, 14.51, 13.28, 11.87; HPLC purity: 95.6%, R_t_ = 8.68 min; HRMS (ESI): calcd for C_50_H_58_ClN_9_NaO_10_S_2_ [M+Na]^+^: 1066.3329, found 1066.3321.

**(*S*)-*N*-(1-(4-(4-chlorophenyl)-2,3,9-trimethyl-6*H*-thieno[3,2-*f*][1,2,4]triazolo[4,3-*a*][1,4]diazepin-6-yl)-2-oxo-6,9,12,15,18,21-hexaoxa-3-azatricosan-23-yl)-3-(*N*-(3-cyano-4-methyl-1*H*-7-indolyl) sulfamoyl)benzamide (Compound 12).** ^1^H NMR (500 MHz, DMSO-*d*_6_) δ 11.98 (d, *J* = 3.2 Hz, 1H), 10.02 (s, 1H), 8.76 (t, *J* = 5.6 Hz, 1H), 8.28 (t, *J* = 5.7 Hz, 1H), 8.24 (s, 1H), 8.17 (d, *J* = 2.9 Hz, 1H), 8.07 (d, *J* = 7.8 Hz, 1H), 7.79 (d, *J* = 8.2 Hz, 1H), 7.65 – 7.58 (m, 1H), 7.49 (d, *J* = 8.7 Hz, 2H), 7.42 (d, *J* = 8.5 Hz, 2H), 6.76 (d, *J* = 7.9 Hz, 1H), 6.52 (d, *J* = 7.7 Hz, 1H), 4.51 (dd, *J* = 8.1, 6.0 Hz, 1H), 3.59 – 3.17 (m, 30H), 2.59 (s, 3H), 2.56 (s, 3H), 2.41 (s, 3H), 1.62 (s, 3H); ^13^C NMR (125 MHz, Chloroform-*d*) δ 171.18, 166.04, 164.47, 155.99, 150.63, 139.78, 136.97, 136.65, 135.55, 133.63, 132.52, 132.12, 131.37, 131.03, 130.75, 130.68, 130.09, 129.88, 129.31, 128.90, 128.77, 127.00, 125.37, 123.11, 120.18, 119.80, 117.62, 86.32, 70.59, 70.51, 70.48, 70.43, 70.40, 70.37, 70.32, 70.17, 69.99, 69.89, 54.24, 40.25, 39.68, 38.86, 18.36, 14.53, 13.28, 11.94. HPLC purity: 96.4%, R_t_ = 8.79 min; HRMS (ESI): calcd for C_50_H_58_ClN_9_NaO_10_S_2_ [M+Na]^+^: 1066.3329, found 1066.3323.

**The synthetic method of DP1(R) is similar to DP1(Compound 4), except using (*R*)-2-(4-(4-chlorophenyl)-2,3,9-trimethyl-6*H*-thieno[3,2-*f*][1,2,4]triazolo[4,3-*a*][1,4]diazepin-6-yl)acetic acid.**

**(*R*)-*N*-(2-(2-(2-(2-(4-(4-chlorophenyl)-2,3,9-trimethyl-6*H*-thieno[3,2-*f*][1,2,4]triazolo[4,3-*a*] [1,4]diazepin-6-yl)acetamido)ethoxy)ethoxy)ethyl)-3-(*N*-(3-cyano-4-methyl-1*H*-indol-7-yl)-sulfamoyl)benzamide (DP1(R)).** ^1^H NMR (500 MHz, DMSO-*d*_6_) δ 11.99 (d, *J* = 3.2 Hz, 1H), 10.03 (s, 1H), 8.77 (t, *J* = 5.6 Hz, 1H), 8.28 (t, *J* = 5.7 Hz, 1H), 8.24 (s, 1H), 8.18 (d, *J* = 3.1 Hz, 1H), 8.08 (d, *J* = 7.8 Hz, 1H), 7.78 (d, *J* = 7.8 Hz, 1H), 7.64 – 7.59 (m, 1H), 7.48 (d, *J* = 8.4 Hz, 2H), 7.42 (d, *J* = 8.3 Hz, 2H), 6.76 (d, *J* = 7.8 Hz, 1H), 6.52 (d, *J* = 7.7 Hz, 1H), 4.51 (dd, *J* = 7.9, 6.2 Hz, 1H), 3.61 – 3.39 (m, 10H), 3.32 – 3.19 (m, 4H), 2.59 (s, 3H), 2.56 (s, 3H), 2.40 (s, 3H), 1.61 (s, 3H); ^13^C NMR (125 MHz, Chloroform-*d*) δ 171.42, 165.99, 165.02, 155.83, 150.86, 139.53, 137.16, 136.37, 135.41, 133.14, 132.76, 132.08, 131.64, 131.03, 130.66, 130.27, 130.03, 129.99, 129.25, 128.96, 128.84, 126.96, 124.68, 123.19, 119.72, 119.67, 117.41, 86.59, 70.46, 69.94, 69.64, 69.56, 54.33, 40.15, 39.76, 39.05, 18.31, 14.49, 13.27, 11.88. HPLC purity: 91.8%, R_t_ = 7.84 min; HRMS (ESI): calcd for C_42_H_42_ClN_9_NaO_6_S_2_ [M+Na]^+^: 890.2280, found 890.2295

.

**The synthetic method of *tert*-butyl(*S*)-2-(4-(4-chlorophenyl)-2,3,9-trimethyl-6*H*-thieno[3,2-*f*]- [1,2,4]triazolo[4,3-*a*][1,4]diazepin-6-yl)acetate (JQ1).**

To a mixture of (*S*)-2-(4-(4-chlorophenyl)-2,3,9-trimethyl-6H-thieno[3,2-*f*] [1,2, 4]triazolo[4,3-*a*][1,4]diazepin-6-yl)acetic acid (40 mg, 0.10 mmol, 1.0 equiv.) and (Boc)_2_O (33 mg, 0.15 mmol, 1.5 equiv.) in 2 mL of dry *tert*-butanol was added DMAP (3 mg). The mixture was stirred at room temperature for 12 h, then removed solvent *tert*-butanol in vacuum got crude product. The crude product was purified by column chromatography (DCM/MeOH = 80/1) to get 29 mg (63% yield) *tert*-butyl(*S*)-2-(4-(4-chlorophenyl)-2,3,9-trimethyl-6*H*-thieno[3,2-*f*][1,2,4]triazolo[4,3-*a*]-[1,4]diazepin-6-yl)acetate **(JQ1).** ^1^H NMR (500 MHz, DMSO-*d*_6_) δ 7.50 (d, *J* = 8.3 Hz, 2H), 7.43 (d, *J* = 8.2 Hz, 2H), 4.42 (dd, *J* = 8.2, 6.2 Hz, 1H), 3.34 (m, 2H), 2.60 (s, 3H), 2.42 (s, 3H), 1.63 (s, 3H), 1.42 (s, 9H); ^13^C NMR (150 MHz, Chloroform-*d*) δ 171.08, 163.89, 155.76, 150.07, 136.90, 131.15, 130.92, 130.67, 130.37, 128.95, 81.25, 54.18, 38.13, 28.41, 16.61, 13.53, 12.18.HPLC purity: 98.3%, R_t_ = 9.27 min; HRMS (ESI): calcd for C_23_H_26_ClN_4_O_2_S[M+H]^+^:457.1460, found 457.1442.


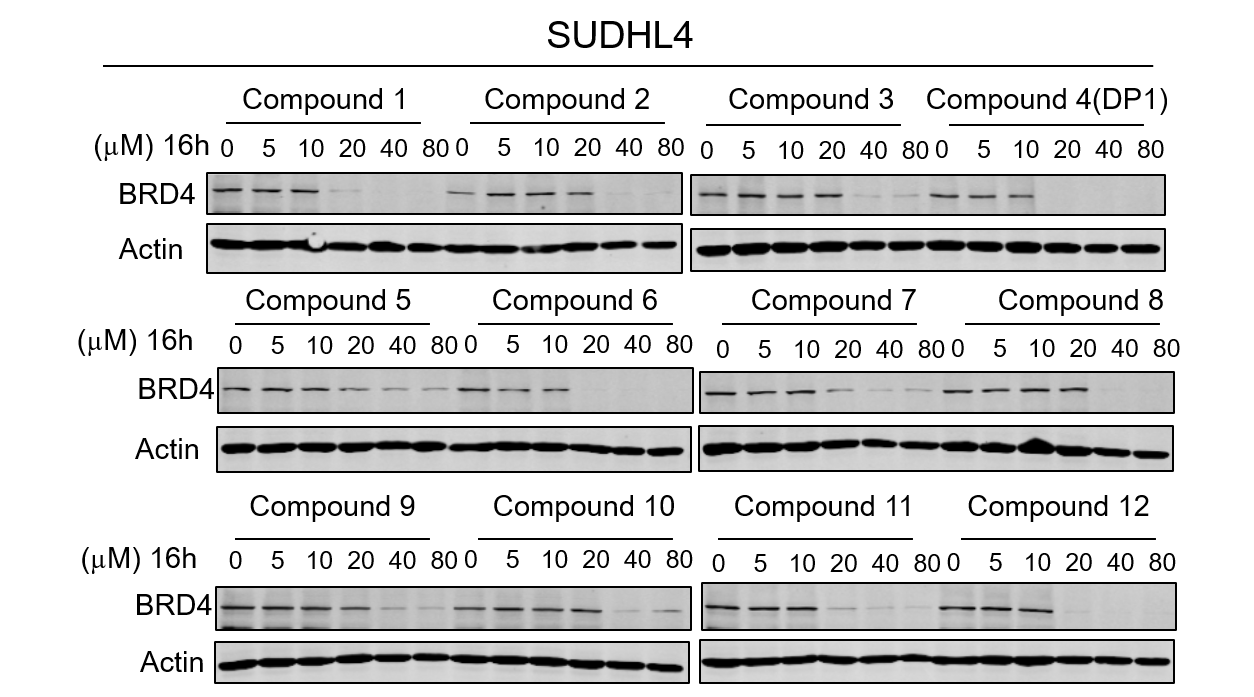
**Figure. S1**

**Figure S1** BRD4 degradation efficacy of twelve PROTACs based on DCAF15 in SU-DHL-4 cell (associated with Table S1).

**Figure. S2**


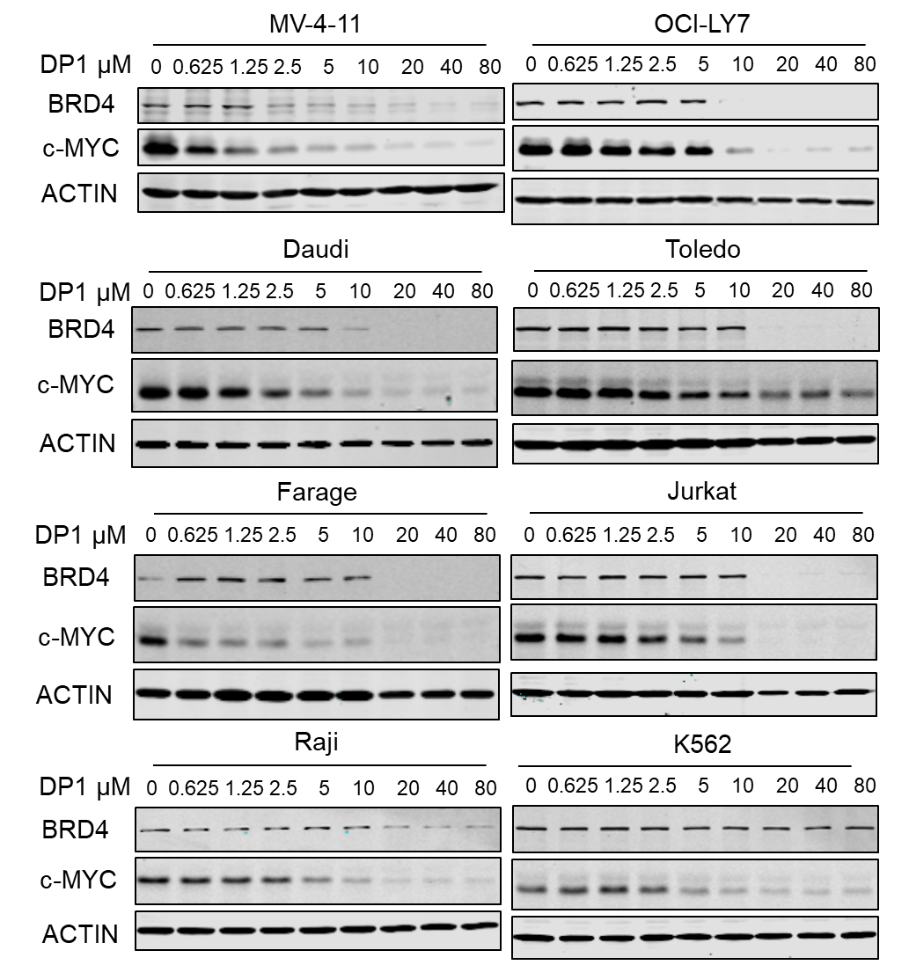


**Figure S2** BRD4 degradation mediated by DP1 is evaluated in various cell lines (associated with Table S2).

**Figure. S3**


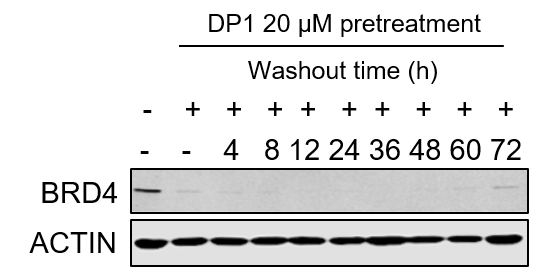


**Figure S3** After 24 h treatment of SU-DHL-4 cells with 20 μM DP1, cells were then washed with PBS for three times and resuspended in fresh media without PROTACs. Protein levels of BRD4 were monitored at the indicated time points.

**Figure. S4**





**Figure S4** *mRNA* levels of *BRD2/3/4* and *c-MYC* after an 16 h treatment of SU-DHL-4 cells with DMSO, 1 μM, 5 μM and 20 μM DP1.

**Figure. S5**


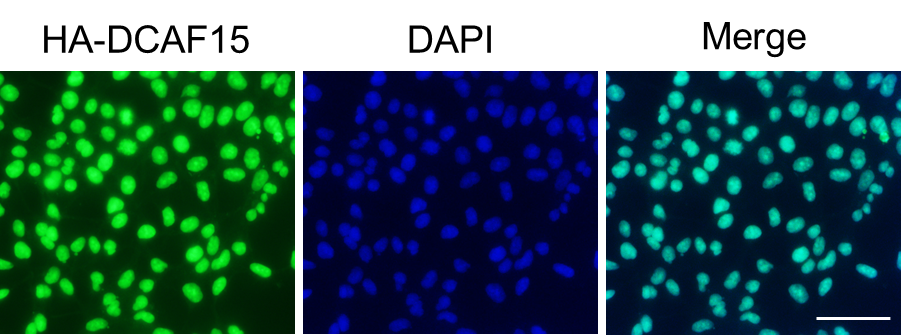


**Figure S5** Immunofluorescence using an anti-HA antibody showing nuclear localization of HA-DCAF15 (expressed by transient transfection in 293T cells). Scale bar, 50 μm. Representative images were collected from two experiments.

**Figure. S6**


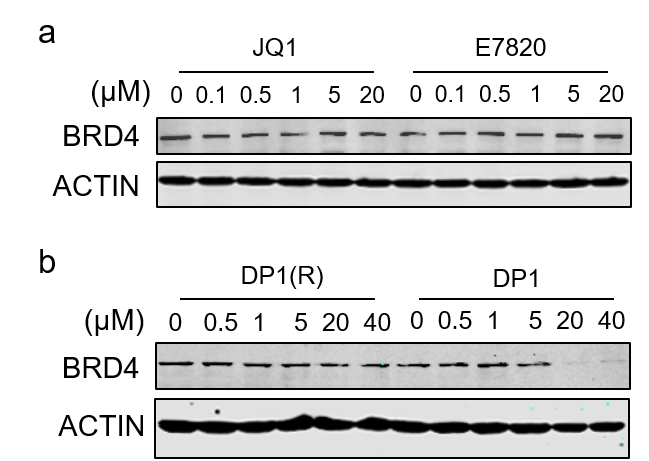


**Figure S6 a** Immunoblot of BRD4 and ACTIN after treatment with the indicated concentrations of JQ1 and E7820 for 24 h. **b** Immunoblot of BRD4 and ACTIN after treatment with the indicated concentrations of DP1 or DP1(R) for 18 h in SU-DHL-4 cells.

**Figure. S7**


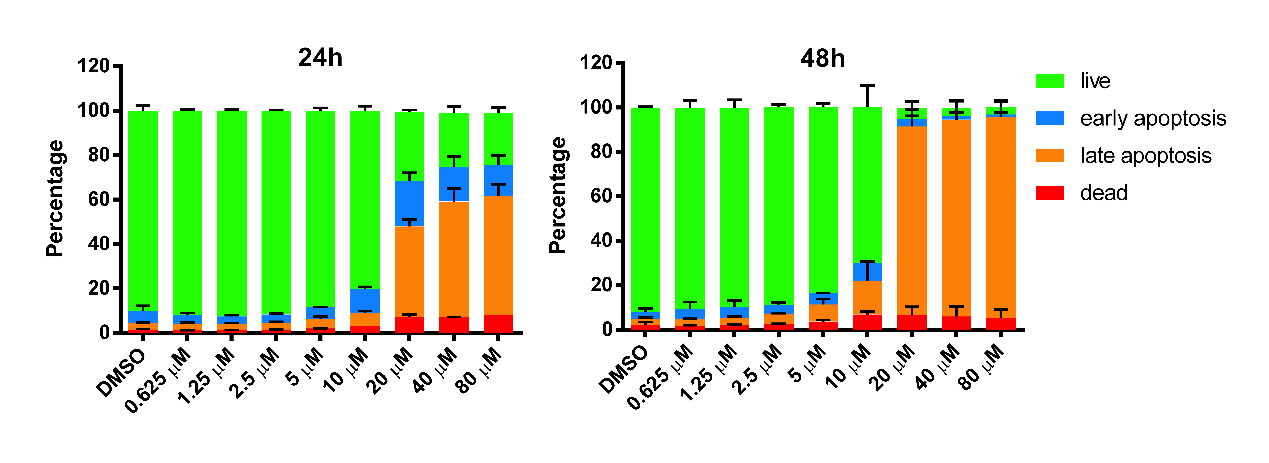


**Figure S7** Flow cytometry analysis of apoptosis of SU-DHL-4 cells treated with the indicated concentrations of DP1 for 12 h (left) and 24 h (right).

**Figure. S8**


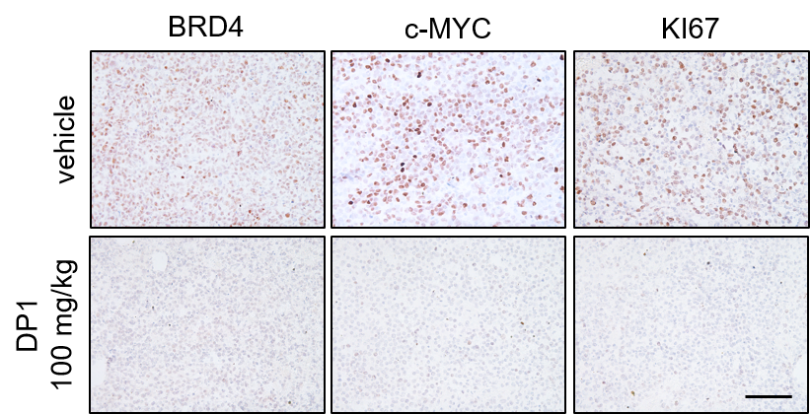


**Figure S8** Immunohistochemistry for BRD4, c-MYC and Ki67 of tumor sections from mice treated with vehicle and DP1. Scale bar, 50 μm. Representative images were collected from three experiments.


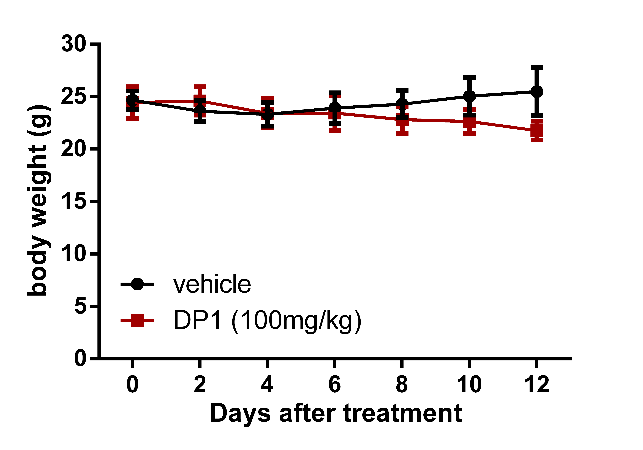
**Figure. S9**

**Figure S9** Weight change of the SCID mice after 12 days treatment with 100 mg/kg intraperitoneal injection of DP1 (n=6).

**Table. S1**

| **Compound** | **DC50 (μM)** | **Dmax** |
| --- | --- | --- |
| **1** | 13.22 ± 0.44 | 90% |
| **2** | 19.85 ± 0.11 | 82% |
| **3** | 34.09 ± 2.5 | 75% |
| **4 (DP1)** | 10.84 ± 0.92 | 98% |
| **5** | 27.69 ± 1.14 | 69% |
| **6** | 11.37 ± 0.63 | 94% |
| **7** | 14.41 ± 0.13 | 86% |
| **8** | 39.00 ± 1.39 | 88% |
| **9** | 26.09 ± 0.24 | 75% |
| **10** | 47.82 ± 8.83 | 71% |
| **11** | 10.93 ± 0.32 | 89% |
| **12** | 18.97 ± 0.13 | 86% |

**Table S1** Degradation efficacy of twelve BRD4 degraders in SU-DHL-4 cell (associated with Figure S1).

**Table. S2**

| **Com**  **DC_50_/D_max_**  **Cell** | **DP1** | |
| --- | --- | --- |
|  | DC_50_(μM) | D_max_ |
| **MV-4-11** | 1.78 ± 0.64 | 95% |
| **OCI-LY7** | 4.96 ± 0.27 | 98% |
| **Daudi** | 5.06 ± 0.51 | 88% |
| **Toledo** | 9.02 ± 0.82 | 90% |
| **SU-DHL-4** | 10.84 ± 0.92 | 98% |
| **Farage** | 13.67 ± 2.94 | 91% |
| **Jurkat** | 13.02 ± 2.07 | 90% |
| **Raji** | 26.01 ± 0.79 | 80% |
| **K562** | >80 | n.d. |

**Table S2** Degradation efficacy of DP1 in different cell lines (associated with Figure S2).

**Table. S3**

| c-MYC-F | TACAACACCCGAGCAAGGAC |
| --- | --- |
| c-MYC-R | GAGGCTGCTGGTTTTCCACT |
| BRD2-F | GCCCTACACCATTAAGAAGCCT |
| BRD2-R | GCTAAGGCGTGACACTGCTA |
| BRD3-F | ACATCATCCAATCTCGGGAGC |
| BRD3-R | CTTCCCGCTTGCTGAGAACG |
| BRD4-F | TGCACATCATCCAGTCACGG |
| BRD4-R | TGCACATCATCCAGTCACGG |

**Table S3** qRT-PCR primers used in experiments.
